# Sleep Quality and Electroencephalogram Delta Power

**DOI:** 10.3389/fnins.2021.803507

**Published:** 2021-12-15

**Authors:** Siyu Long, Rui Ding, Junce Wang, Yue Yu, Jing Lu, Dezhong Yao

**Affiliations:** ^1^MOE Key Lab for Neuroinformation, The Clinical Hospital of Chengdu Brain Science Institute, University of Electronic Science and Technology of China, Chengdu, China; ^2^School of Life Sciences and Technology, Center for Information in Medicine, University of Electronic Science and Technology of China, Chengdu, China

**Keywords:** delta power, sleep quality, EEG, sleep stage, sleep disorder

## Abstract

Delta activity on electroencephalogram (EEG) is considered a biomarker of homeostatic sleep drive. Delta power is often associated with sleep duration and intensity. Here, we reviewed the literature to explore how sleep quality was influenced by changes in delta power. However, we found that both the decrease and increase in delta power could indicate a higher sleep quality due to the various factors below. First, the differences in changes in delta power in patients whose sleep quality is lower than that of the healthy controls may be related to the different diseases they suffered from. We found that the patients mainly suffered from borderline personality disorder, and Rett syndrome may have a higher delta power than healthy individuals. Meanwhile, patients who are affected by Asperger syndrome, respiratory failure, chronic fatigue, and post-traumatic stress disorder have lower delta power. Second, if the insomnia patients received the therapy, the difference may be caused by the treatment method. Cognitive or music therapy shows that a better therapeutic effect is associated with decreased delta power, whereas in drug treatment, there is an opposite change in delta power. Last, for healthy people, the difference in delta change may be related to sleep stages. The higher sleep quality is associated with increased delta power during the NREM period, whereas a deceased delta change accompanies higher sleep quality during the REM period. Our work summarizes the effect of changes in delta power on sleep quality and may positively impact the monitoring and intervention of sleep quality.

Delta oscillation reflects the burst-pause firing pattern of the hyperpolarized thalamic cortex and corticothalamic neurons in synchronization (Steriade et al., 1993). Delta activity can be measured by delta power by Fourier analysis. It is a biomarker of homeostatic sleep drive, as evidence shows that delta power is enhanced after prolonged wakefulness, but declines as sleep deepens (Feinberg, 1974; Borbély et al., 1981; Achermann and Borbély, 2011). One study showed that after 40 h of sleep deprivation, both young and old subjects showed a significant increase in the power spectral density in the delta band (Münch et al., 2004). The finding that delta power is high during wake time but decreases during sleep (Esteban et al., 2005) was supported by healthy participants from various age groups as well as sleep-deprived people. A study of infants, adolescents, and adults showed that both children and adults experienced a gradual decrease in delta power and slow-wave sleep throughout the night (Bes et al., 1991). Sleep analysis of sleep-deprived young adults showed the gradual decline of electroencephalogram (EEG) power density in the low-frequency range as an indicator during sleep (Borbély et al., 1981). In addition, delta power is often accompanied by sleep duration and intensity (Davis et al., 2011). For example, after a lack of sleep, EEG delta power and non-rapid eye movement (NREM) duration during NREM episodes will increase (Pappenheimer et al., 1975; Achermann, 2004; Davis et al., 2011). Thus, delta power is a crucial part of the sleep process. We review some literature related to the change in delta power influencing sleep quality. However, current research on the relationship between delta power and sleep quality is inconclusive (Gao et al., 2020; Johnson and Durrant, 2021).

There is a lack of analysis on which factors lead to these opposite aspects. We list out the studies related to the change in delta power, including the advantages of decreased delta power and increased delta power (Tables 1, 2). We summarize the factors that may influence the adverse delta change. A wide range of factors, including the type, age, number, and sex of participants, the period of delta power changes, EEG derivation, and the power calculation method, may result in different conclusion.

**TABLE 1:** A delta power decrease indicates better sleep in different situations.

	**Participants type**	**Sex**	**Participants amount**	**Age**	**Period^a^**	**EEG derivations**	**Calculation index**	**EEG duration per epoch**	**Filter**	**FFT window length**	**Results**
Philipsen et al.,2005	borderline personality disorder (BPD)/healthy	Female/female	20/20	28.6 ± 7.88/ matched	All night	C3-A2 and C4-A1	power spectrum	30 s	70 Hz low-pass	512 points (2.56 s)	Patients with BDP had lower NREM sleep and showed increased delta power throughout NREM and REM.
Lindberg et al.,2003	patients with highly violent offences	Male	16	30.75 ± 10.46	NREM	O2-P4 and O1-P3	power spectrum	Not mentioned	0.2–25 Hz band-pass	Not mentioned	The patients who suffered from BPD have higher delta power and lower sleep quality than the control group.
Buysse et al.,2008	primary insomnia (PI)/good sleeper controls (GSC)	Mixed^b^/Mixed^b^	48/25	30.8 ± 7.2/30.6 ± 7.4	NREM	bilateral central EEG leads	power spectrum	20 s	0.3–100 Hz band-pass (60 Hz notch)	4 s	The patients with primary insomnia have higher delta power compared with a good sleeper.
Ammanuel et al.,2015	Rett syndrome (RTT)/non-RTT	Female/female	10/15	2–9	SWS	F3, C3, O1	power spectrum	10 s	1–70 Hz band-pass	Not mentioned	The patients with RTT have higher delta power, accompanied by lower sleep efficiency.
Münch et al.,2004	healthy	Mixed^b^	16/16	25 ± 0.9/64.9 ± 1.4	Not mentioned	Fz, Cz, Pz, Oz	power density	20 s	30 Hz low-pass	4 s	After sleep deprivation, the delta power density of the youth and the old are both increased.
Gabryelska et al.’s,2019	healthy	Mixed^b^	206	19–73	REM	C3-A2 and C4-A1	power density	30 s	Not mentioned	256 points (2.5 s) for 102.4 Hz sample rate and 512 points (2.56 s) for 200 Hz	Better sleep quality is significantly associated with decreased Delta 1 power density in REM.
Borbély et al.,1981	healthy	Mixed^b^	8	21–29	NREM- REM sleep cycles	ipsilateral fronto-occipital bipolar derivation	power density	30 s	25 Hz low-pass	4 s	During NREM-REM sleep cycles, delta power density declines.
Dijk et al.,1990	healthy	Male	9	22–26	NREM-REM sleep cycles	C3-A2 and C4-Al	power density	20 s	25 Hz low-pass	4 s	Delta power spectrum decreased during the first three and four NREM-REM cycles.
Ehlers and Kupfer,1989	healthy	Male	24	21–70	NREM	C4	power density	60 s	0.3–30 Hz band-pass	4 s	The largest decrease in delta activity occurred to the greatest extent during the first 100 min of sleep (NREM period 1).
Krystal and Edinger,2010	primary insomnia patients (PI)	Mixed^b^	30	40–80	NREM	C3-M2 and Oz-Cz	power spectrum	30 s	0.5–64 Hz band-pass	2 s	Patients treated with CBT (cognitive behavior treatment) has a faster decrease in delta power and a better self-assessment of sleep quality.

*^a^Period, The period during which delta changes.*

*^b^Mixed-gender group of male and female.*

**TABLE 2:** A delta power increase indicates better sleep in different situations.

	**Participants type**	**Sex**	**Participants amount**	**Age**	**Period^a^**	**EEG derivations**	**Calculation index**	**EEG duration per epoch**	**Filter**	**FFT window length**	**Results**
Cohen et al.,2018	preterm fetal growth restricted (FGR) neonates/preterm appropriate-for-gestational-age (AGA) peers/healthy	Not mentioned	18/20/19	1–6 mAVonths	Not mentioned	C3-M2 and O1-M2 or C4-M1 and O2-M1	power spectrum	10 s	0.5–30 Hz band-pass	4 s	The delta power of p-FGR was significantly reduced compared to p-AGA infants.
Tani et al.’s,2004	Asperger syndrome/healthy	Not mentioned	20/10	27.2 ± 7.3/26.5 ± 8.1	SWS	C3-A1 and C4-A1	power spectrum	30 s	0.5–45 Hz band-pass	512 points (5.12 s)	A statistically non-significant trend toward decreased relative delta power and increased theta power in slow-wave sleep was found in the AS group.
Kudchadkar et al.,2015	children with respiratory failure/healthy	Not mentioned	8/unknown	6–16/unknown	Not mentioned	bilateral central and occipital EEG leads	power spectrum	30 s	Not mentioned	5 s	Differences noted included significantly lower mean nighttime delta power in the PICU patients compared to healthy children.
Wang et al.,2020	PTSD patient/non-PTSD	Mixed	31/47	31.3 ± 4.7/32.8 ± 6.2	NREM	Not mentioned	power spectrum	5 s	0.5–50 Hz band-pass	5 s	Compared with non-PTSD, PTSD exhibited reduced delta power during NREM sleep.
Monti et al.’s,2000	insomniac patients	Female	12	27–59	Not mentioned	Not mentioned	power density	20 s	0.25–30 Hz band-pass	Not mentioned	All-night spectral analysis of the EEG revealed that power density in NREM sleep was significantly increased in the low-frequency band (0.25–1.0 Hz) in the zolpidem group during the first 2-h interval.
Hall et al.,2000	healthy	Mixed^b^	14	53 ± 12	NREM	C3 or C4	power density	60 s	Not mentioned	4 s	Increases in subjective stress burden were associated with decreases in delta power.
Perrier et al.,2015	primary insomnia/healthy	Mixed^b^/Mixed^b^	14/10	47 ± 17/46 ± 15	NREM	FP1, FP2 C3, C4, O1, O2, T3, T4,	power spectrum	Not mentioned	0.16–70 Hz band-pass	5.12 s	Primary insomnia patients exhibited a lower delta power spectrum compared to good sleepers.
Guilleminault et al.’s,2006	chronic fatigue/healthy	Mixed^b^/Mixed^b^	14/14	41.1 ± 9.8/33.6 ± 10.2	SWS	C4-A1	power spectrum	60 s	Not mentioned	4 s	Slow-wave sleep (SWS) percentage and sleep efficiency were lower, but there was a significant decrease in delta 2 relative power in the chronic fatigue group when compared to normals.
Kuula et al.’s,2020	healthy	Mixed^b^	20	24.5 ± 3.5	NREM	F3, F4, C3, C4, O3, O4, A1, and A2	power density	30 s	0.5–35 Hz band-pass	1024 points (4 s)	In the slow breathing condition (sleep quality is higher), higher central delta power during N3 was observed.
Park et al.’s,2021	healthy	Men	9	23.8 ± 0.7	SWS (N3)	C3-A2, C4-A1, O2-A1, O1-A2, F3-A2, F4-A1	power spectrum	30 s	Not mentioned	5 s	The group which experienced exercise shows significantly increased delta power in SWS associated with increased SWS stability and short REM latency.
Hoch et al.,2001	healthy	Mixed^b^	21	70–91	Not mentioned	Not mentioned	Not mentioned	Not mentioned	0.3–30 Hz band-pass	Not mentioned	Participants in the bed-restriction group showed a median increase in sleep efficiency of 6.1 versus 1.8% in participants receiving sleep hygiene instruction and an increase in all-night delta EEG power.
Manconi et al.,2017	adults with a diagnosis of chronic primary insomnia/healthy	Not mentioned	20/13	Not mentioned	NREM and SWS	C3-A2 or C4-A1	power spectrum	2 s	0.1–35 Hz band-pass	2 s	During NREM sleep, patients showed a clear decrease in the relative power of the delta band.
Izci-Balserak et al.,2018	pregnancy	Female	123	27.15 ± 7.2	NREM	C3-A2 and C4-A1	power spectrum	30 s	0.3–100 Hz band-pass (60 Hz notch)	4 s	In late pregnancy, women had shorter sleep duration, poorer sleep efficiency, more awakenings, more stage N2 sleep, less slow-wave sleep, less REM sleep, higher AHI, and a higher periodic limb movement index than early pregnancy. Delta and theta powers decreased.

*^*a*^Period, The period during which delta changes.*

*^*b*^Mixed-gender group of male and female.*

We summarize the factors that may have influenced the contrary results. First, we infer that the category of the disease may influence the quality of sleep from various aspects, which may have different impacts on EEG power. We found that the patients in Table 1 mainly suffered from borderline personality disorder (BPD), Rett syndrome (RTT) (Lindberg et al., 2003; Philipsen et al., 2005; Ammanuel et al., 2015). A study of female patients with BPD who often have trouble sleeping showed that the delta power of BPD patients increased during NREM and REM sleep. Meanwhile, subjective scores, which mainly use the Pittsburgh Sleep Quality Index (PSQI) (Buysse et al., 1989), recorded a significant decline in sleep quality among BPD patients (Philipsen et al., 2005). It has also been suggested that sleep problems that present as reduced sleep duration, a longer incubation period, and interrupted sleep in girls (2–9 years old) with RTT were associated with higher delta frequency (Ammanuel et al., 2015). However, Table 2 mainly shows the patients who were affected by Asperger syndrome, respiratory failure, chronic fatigue, and post-traumatic stress disorder (PTSD) (Tani et al.’s, 2004; Guilleminault et al.’s, 2006; Kudchadkar et al., 2015; Wang et al., 2020). Tani et al.’s (2004) research mainly compared patients suffering from Asperger syndrome (27.2 ± 7.3) with healthy individuals and found a decreased delta power spectrum trend in patients during the SWS stage. The patients also had subjective impairment in the initiation and continuity of sleep. A study showed that children with respiratory failure who were associated with disruption of the normal sleep-wake rhythm had lower delta power than healthy children (Kudchadkar et al., 2015). Guilleminault et al.’s (2006) study compared the difference between chronic fatigue and normal and found that slow-wave sleep (SWS) percentage and sleep efficiency were lower, but there was a significant decrease in delta 2 relative power in the chronic fatigue group when compared to normal. Wang et al.’s (2020) study showed that compared with non-PTSD patients, PTSD patients exhibited reduced delta power during NREM sleep with reduced sleep depth. Second, the difference may be related to the method of treatment. Different therapies influence sleep quality in diverse ways. In studies of insomnia patients treated by cognitive behavioral therapy (CBT), the results showed that the continuous decrease in delta power was related to the treatment effect, and the rate of decrease in delta power was also used as the criterion of sleep drive (Feinberg, 1974; Krystal and Edinger, 2010). In addition, our previous study also found that SWS brain-wave music (Wu et al., 2009; Lu et al., 2012; Yao et al., 2020) leads to shorter sleep latency and higher sleep efficiency with decreased delta power (Gao et al., 2020). However, in Monti et al.’s (2000) study, power density in NREM sleep increased significantly in the low-frequency band (0.25–1.0 Hz) in the zolpidem group. We found that patients who received cognitive/music treatment and drug therapy had adverse delta changes (Monti et al.’s, 2000; Krystal and Edinger, 2010). Last, the period of delta change may influence the final results. We found from healthy participants that when the change in delta power occurred in the NREM period, better sleep quality was associated with increased delta power, which was proven in Kuula et al.’s (2020) study. They found that participants who received slow breathing training with higher sleep quality had higher delta power during N3, which is a deep sleep stage that lasts approximately 20–40 min. There is also evidence showing that increases in the subjective stress burden were associated with decreases in delta power during NREM (Hall et al., 2000). Research in Park et al.’s (2021) found that exercise can lead to a short rapid eye movement latency and significantly increased delta power in SWS with enhanced SWS stability in early sleep phases. However, Gabryelska et al.’s (2019) study showed different situations in which better sleep quality was significantly associated with decreased Delta 1 power density in REM. Considering this fact, we speculate that the period of delta change may influence the impact of delta change.

Besides, we also listed some parameters about the delta power spectrum processing (Tables 1, 2) and wondered if these factors include the EEG duration per epoch, filter frequency, and the fast Fourier transform (FFT) window length might affect the final results. However, we did not find the common characteristic in each condition. Therefore, these parameters do not influence the relationship between delta power change and sleep quality.

The delta power is like a marker whose change can detect the sleep quality. The current research we have conducted mainly speculated the possible reason for the increase or the decrease of delta power indicates the better sleep quality. However, they are some speculations that we conclude by comparing the different studies. In the future, we should further explore the physiology mechanism behind these factors influencing the change of delta power and further influence sleep quality, which will provide more reliable evidence for the current study and draw a more precise conclusion on how the shift in delta power influences sleep quality.

In summary, we summarize the studies related to the change in delta power associated with sleep quality. We inferred that some factors might influence the difference in the impact of changes in delta power. We hope our research may contribute to a deeper understanding of the impact of delta power on monitoring and intervention of sleep quality.

## Author Contributions

SL, RD, JL, and DY conceived the study and drafted the manuscript. JW and YY collected the information. All authors made critical revisions to the work, which ultimately approved the upcoming edition, and agreed to be responsible for all aspects of the work.

## Conflict of Interest

The authors declare that the research was conducted in the absence of any commercial or financial relationships that could be construed as a potential conflict of interest.

## Publisher’s Note

All claims expressed in this article are solely those of the authors and do not necessarily represent those of their affiliated organizations, or those of the publisher, the editors and the reviewers. Any product that may be evaluated in this article, or claim that may be made by its manufacturer, is not guaranteed or endorsed by the publisher.
